# c-kit+ cells offer hopes in ameliorating asthmatic pathologies via regulation of miRNA-133 and miRNA-126 

**DOI:** 10.22038/ijbms.2021.49008.11231

**Published:** 2021-03

**Authors:** Reza Rahbarghazi, Rana Keyhanmanesh, Jafar Rezaie, Fatemeh Mirershadi, Hossain Heiran, Hesam Saghaei Bagheri, Shirin Saberianpour, Aysa Rezabakhsh, Aref Delkhosh, Yasin Bagheri, Hadi Rajabi, Mahdi Ahmadi

**Affiliations:** 1 Stem Cell Research Center, Tabriz University of Medical Sciences, Tabriz, Iran; 2 Department of Applied Cell Sciences, Faculty of Advanced Medical Sciences, Tabriz University of Medical Sciences, Tabriz, Iran; 3 Tuberculosis and Lung Diseases Research Center, Tabriz University of Medical Sciences, Tabriz, Iran; 4 Solid Tumor Research Center, Research Institute for Cellular and Molecular Medicine, Urmia University of Medical Sciences, Urmia, Iran; 5 Department of Physiology, Ardabil Branch, Islamic Azad University, Ardabil, Iran; 6 Department of Physiology, Faculty of Medicine, Tabriz University of Medical Sciences, Tabriz, Iran; 7 Vascular and Endovascular Surgery Research Center, Mashhad University of Medical Science, Mashhad, Iran; 8 Physical Medicine and Rehabilitation Research Center, Aging Research Institute, Tabriz University of Medical Sciences, Tabriz, Iran; 9 Young Researchers and Elite Club, Tabriz Branch Islamic Azad university, Tabriz, Iran

**Keywords:** Cell therapy, Histological changes, Lung, Ovalbumin, Rat

## Abstract

**Objective(s):**

There are still challenges regarding c-kit+ cells’ therapeutic outcome in the clinical setting. Here, we examined the c-kit+ cell effect on the alleviation of asthma by modulating miRNAs expression.

**Materials and Methods:**

To induce asthma, male rats were exposed to ovalbumin. Bone marrow-derived c-kit+ cells were enriched by MACS. Animals were classified into four groups (6 rats each). Control rats received PBS intratracheally; Ovalbumin-sensitized rats received PBS intratracheally; Ovalbumin-sensitized rats received PBS intratracheally containing 3×105 c-kit+ and c-kit- cells. Cells were stained with Dil fluorescent dye to track in vivo condition. Pathological changes were monitored in asthmatic rats after transplantation of c-kit+ and c-kit- cells. Serum levels of IL-4 and INF-γ were measured by ELISA. Transcription of miRNAs (-126 and 133) was assessed by real-time PCR analysis.

**Results:**

Pathological examination and Th1 and Th2 associated cytokine fluctuation confirmed the occurrence of asthma in rats indicated by chronic changes and prominent inflammation compared with the control group (*P*<0.05). Both c-kit+ and c-kit- cells were verified in pulmonary niche. Administration of c-kit positive cells had the potential to change INF-γ/IL-4 ratio close to the normal values compared with matched-control asthmatic rats (*P*<0.05). We also found that c-kit+ cells regulated the expression of miRNA-126 and -133, indicated by an increase of miRNA-133 and decrease of miRNA-126 compared with cell-free sensitized groups (*P*<0.05).

**Conclusion:**

c-kit- cells were unable to promote any therapeutic outcomes in the asthmatic milieu. c-kit+ cells had the potential to diminish asthma-related pathologies presumably by controlling the transcription of miRNA-126 and -133.

## Introduction

Despite advances in human medicine, there are some pathological conditions without definite evidence of therapeutic approach. Among them, asthma is highly prevalent in all parts of the world, ranging from 0.2 to 21.0%, with considerable healthcare costs (1, 2). The current strategies on asthmatic subjects rely on the modulation of inflammatory responses by the application of pharmacological agents. However, these agents are not applicable for a long period and are conceived as symptomatic treatments to alleviate or suppress asthmatic attacks due to commonly reported side effects and inefficiency to alleviate aberrant remodeling (2, 3). Therefore, research targeting pathogenesis and treatment protocols have been a hot topic in the context of the asthmatic milieu. Most of the asthmatic signs are originated from unwanted and recurrent inflammatory response toward exogenous allergens during a long period of exposure(4). Evidence suggests that conversion of Th1/2 ratio to a Th2 type and production of Th2 cytokines in response to inhaled allergens plays a pivotal role in the pathogenesis of asthma (5). Therefore, novel approaches should meet modalities with the ability to change the Th2/Th1 ratio in favor of Th1 (6). Despite the promising effects of multiple stem cell types in the healing and restoration of injured tissues, there is a long way to deciphering optimal cell types with highly efficient outcomes (7, 8). This issue was particularly highlighted when some experiments showed poor stem cell viability and inappropriate engraftment (7). Therefore, the priority issue is to find proof-of-concept observation that directs the application of suitable stem cell types in different injuries. Also, the discovery of underlying mechanisms governed by stem cells helps us to apply these results from animal studies to human medicine (9). Recently, the use of fraction of bone marrow stem cells, namely c-Kit (CD177) positive cells, attracted wide attention for alleviation of different pathologies (8, 10, 11). The term c-Kit stands for a kind of tyrosine kinase receptor that is located on the cell surface and is usually used for the characterization of candidate cells. Due to the existence of heterogeneity in differentiation and therapeutic effects of bone marrow resident c-kit cells, it is logical to hypothesize that diverse therapeutic outcomes will be achieved after administration of these cells into the target sites (8). MiRNAs, small, short (with 19 to 25 nucleotides) non-coding RNAs, exert inhibitory effects at transcriptional and post-transcriptional levels after attachment to the mRNAs 3′UTRs. These regulatory agents participate in different cell bioactivities (12, 13). It has been revealed that the expression of miRNAs is prominently altered in the pulmonary system after the onset of inflammatory cytokine synthesis. However, the critical role of miRNAs has not been neglected in relation to tissue regeneration. According to previous experiments, these genetic elements could be used as biomarkers for the detection of different pathologies (14, 15); even though, the modulating effect of miRNA should be determined to control immune-mediated inflammatory responses (2, 13, 15). Previously, the critical role of both miRNA-126 and miRNA-133 has been diagnosed in animal asthma models. For instance, modulation of miRNA-126 and miRNA-133 was shown to reduce asthmatic remodeling via the inhibition of TH2 inflammatory bioactivity (15, 16). To our knowledge, no documents exist regarding the modulatory effects of c-kit^+^ cells on expression of miRNA-126 and miRNA-133 in asthmatic rats. Therefore, we proposed that administration of c-kit^+^ cells could inhibit/reduce asthmatic changes in the rat model via regulation of miRNA-126 and miRNA-133. 

## Materials and Methods

***Animal ethics***

All phases of this study were fulfilled in accordance with The Care and Use of Laboratory Animals (NIH Publication No. 85- 23, revised 1996) guideline and were reviewed and approved by the Animal Research Ethics Board of Tabriz University of Medical Sciences (IR.TBZMED.VCR.REC.1398.090).

***Experimental animals and group assignment***

**Figure 1:**
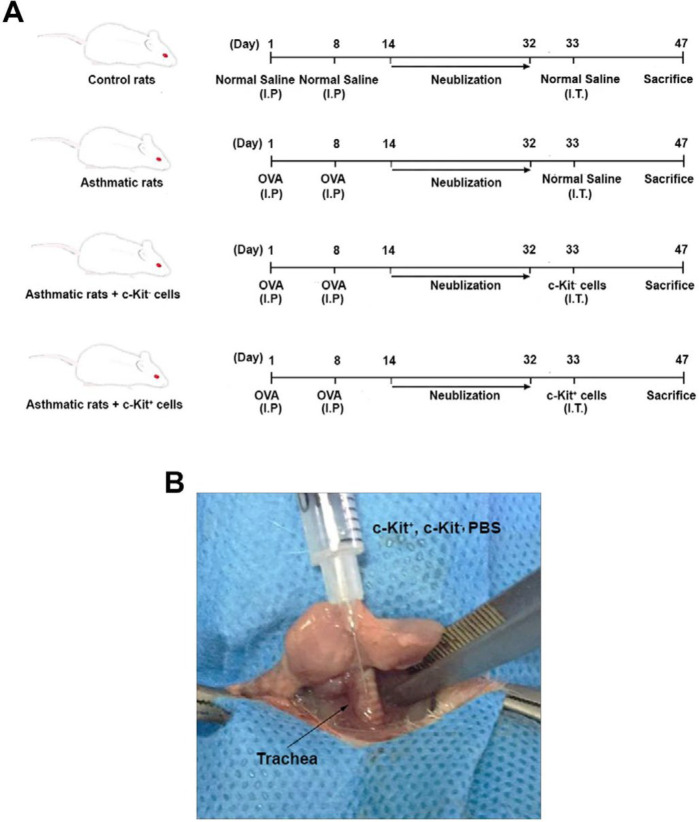
Total scheme of experimental design in this study (A). Intratracheal administration of c-Kit positive and negative cells (B). A total volume of 50 µl samples containing 3×10^5^ cells was injected

The present study was done by enrolling 34 male Wistar rats (8–10 weeks old, weighing 200–250 g). Animals were kept at 22 °C ± 2 °C with 12 hr light/dark cycle. All rats were allowed to access water and rodent pellets freely. After a two-week inhabitation period, 10 rats were blindly selected for isolation of bone marrow content c-Kit^+^ cells. Animals were randomly allocated into four groups (each in 6 rats) as follows: healthy rats only received 50 μl normal saline intratracheally (group C); sensitized rats, received 50 μl normal saline intratracheally (group A); sensitized rats, received 50 μl PBS intratracheally containing 3×10^5^ c-kit^- ^cells (group A+ c-kit^-^); and sensitized rats, received 50 μl normal saline intratracheally containing 3×10^5^ c-kit^+ ^cells (group A+ c-kit^+^) (Figures 1a, b). 

***Animals sensitization protocol***

We used a protocol for a period of 32 ± 1 days for induction of asthmatic changes according to our previous experiment (2). For this propose, rats received 1 ml sterile normal saline containing 1 mg ovalbumin (Sigma -Aldrich, USA) and 200 mg aluminum hydroxide intraperitoneally on days 1 and 8. Six days post intraperitoneal injection, the animals were exposed to aerosolized ovalbumin (4% w/v) from day 14 to 32 ± 1 for 5 min daily using an ultrasonic nebulizer (CX3, Omron Co., Netherlands) connected to a Plexiglas chamber (30 cm × 20 cm × 20 cm). The control rats were treated with saline instead of OVA. After completion of OVA treatment, all rats were anesthetized on day 33, dissected via the ventral neck, and received PBS, c-kit^+^, and c-kit^-^ cells according to group allocation (8, 17). Animals were kept for the next 14 days (17).

***Isolation of c-Kit*** ^+^ ***cells by magnetic activated cell sorting (MACS)***

After cervical dislocation, the upper and lower extremities of the femurs were cut by sterile scissors. Medullary contents were washed by pushing PBS containing 2% fetal bovine serum (FBS; Gibco, USA) using a syringe connected to an 18-gauge needle. Mononuclear cells (MNCs) were collected by gradient centrifugation using Ficoll-Hypaque^®^ solution (Sigma-Aldrich, USA). To this end, cells were centrifuged at 400 *g* for 20 min and monolayer cell located interphase gently collected. Following twice washing with PBS, MNCs were incubated in PBS containing 1% FBS and incubated at 4 °C for 30 min. Then, cells were incubated with mouse-anti human c-Kit microbead (Miltenyi Biotech, Germany) according to the manufacturer’s instructions. By passing cells through the LS columns (Miltenyi Biotec, Germany), c-Kit^+^ and c-Kit^-^ cells were isolated and used for different analyses (18).

***Immunophenotyping of c-kit*** ^+^ ***cells by flow cytometry***

The multipotentiality of isolated cells was studied after MACS by flow cytometry analysis (18). In short, cells in both groups were incubated with FITC-conjugated mouse-anti human CD117 (c-kit^+^) at 4 °C for 30 min. The samples were analyzed by BD FACSCalibur and raw data processed using the FlowJo software package (Ver. 7.6.1). 

***Cell labeling***

Cells were labeled using Cell Tracker^TM^ CM-Dil as previously described (9). Cells were re-suspended in 20 µM Cell Tracker^TM^ CM-Dil solution and kept at 37 °C for 30–40 min. Thereafter, cells were washed with PBS three times (each for 5 min). 50 µl PBS aliquots containing 3×10^5^ c-Kit^+^ and/or c-Kit^-^ cells were prepared. 

***Serum levels of IL-4 and IFN-γ***

Fourteen days after the completion of asthma induction, animals were euthanized by overdose of Ketamine and Xylazine. Blood samples were collected via the inferior vena cava, allowed to clot at RT condition, and serum was harvested by centrifugation at 3000 rpm at 4 °C for 10 min. The serum levels of cytokines IL-4 and IFN-γ were measured using rat ELISA kits (Sigma-Aldrich, USA) according to the manufacturer’s instructions (19). 

***Real-time PCR analysis***

Transcription of miRNA-126 and miRNA-133 was quantitatively measured by conventional real-time PCR assay (9). The Total RNA content was extracted from the left lungs using a total RNA extraction mini kit (Yekta Tajhiz, Iran) and quantified using a NanoDrop ND-1000 spectrophotometer (Thermo Scientific, Wilmington, DE, 19810 USA). Total RNA was transcribed into cDNA using a cDNA synthesis kit (Yekta Tajhiz, Iran). Real-time PCR reaction was done on a Corbett Rotor*-*Gene 3000 instrument (Corbett Life Science, Australia) using SYBR Green master mix (Yekta Tajhiz, Iran). In the current experiment, miRNA-191 was used to normalize miRNA-126 and miRNA-133 values. The data were analyzed in accordance with the 2^−ΔΔCt ^method. Primers used in this study are listed in Table 1.

***Histopathological examination***

Right pulmonary lobes were fixed in 10% neutral buffered formalin solution. Paraffin-embedded samples were cut into the 4-μm thick longitudinal sections by using a Leica microtome. Hematoxylin-Eosin (H&E) and Periodic acid–Schiff (PAS) staining were used to show the pathological changes (2, 20). The existence of chronic pathological features such as hyperemia, emphysema, interstitial pneumonitis, and epithelial cell injury was scored by an independent pathologist. A four-point semi-quantitative score system ranging from 0 to 3 [0: absence, 1: mild injury, i2: moderate injury, and 3 severe injuries] was used to report the extent of pathological changes. 

***Data analysis***

All quantitative results are presented as mean±SEM and analyzed using a one-way ANOVA and Tukey–Kramer *post-hoc* test. Pathological scores were evaluated using the Kruskal-Wallis test followed by *post-hoc* Mann-Whitney analysis. Statistical significance was set at *P*<0.05.

## Results

***Confirmation of c-Kit*** ^+^ ***cell phenotype by flow cytometry***

**Figure 2:**
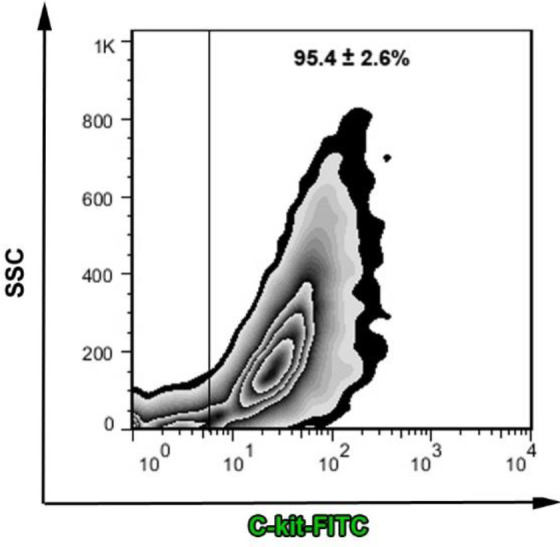
Confirmation of enriched c-kit+ phenotype after MACS procedure. A high rate of pure cells (over 90% desired cell phenotype) was observed after flow cytometry analysis (n=3)

The percent of c-kit^+^ cells was calculated after MACS enrichment by the flow cytometry method. Flow cytometry analysis showed the existence of 95 ± 4.9% c-Kit^+^ cells after enrichment, indicating suitable purification and enrichment of the desired population before transplantation (Figure 2).

***Homing of transplanted cells into the pulmonary niche***

**Figure 3:**
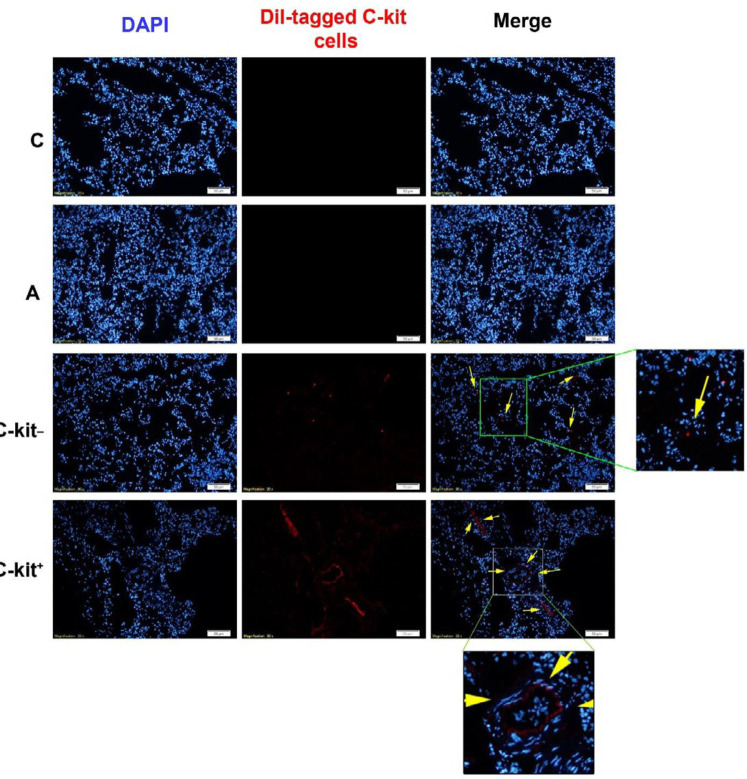
Monitoring the existence of Dil-labeled c-kit+ and c-kit- in pulmonary sections. IF staining revealed the presence of transplanted cells in the pulmonary niche indicated by red-colored appearance. To stain nuclei, we used DAPI stain. Control group (C), sensitized group (A), sensitized animals received c-kit- cells (A + c-kit- group), sensitized animals received c-kit+ cells (A + c-kit+ group) (for each group, n=6)

Immunofluorescence staining showed the presence of red-colored (Dil^+^) cells in the pulmonary tissue, showing the ability of both negative and positive c-kit cells to reach pulmonary tissue via the intra-tracheal route (Figure 3).

***c-kit*** ^+^ ***cells showed the ability to change*** ***the systemic levels of IL-4 and IFN-γ in asthmatic rats***

**Figure 4:**
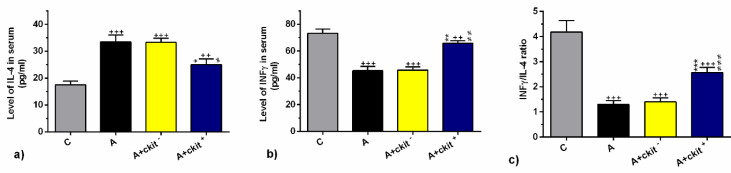
Serum levels of IL-4 (a), IFN-γ (b), and ratio of IFN-γ to IL-4 (c) in control group (C), sensitized group (A), sensitized animals received c-kit- cells (A + c-kit- group), sensitized animals receiving c-kit+ cells (A + c-kit+ group) (for each group, n=6). Bars represent the mean±SEM. Statistical differences between control and different groups: ++; *P*<0.01 and +++; *P*<0.001. Statistical differences between A+ c-kit+ and A+ c-kit- vs A group: *; *P*<0.05, **; *P*<0.01 and ***; *P*<0. 001. Statistical differences between A+ c-kit+ and A+ c-kit- groups: #; *P*<0.05, ##; *P*<0.01, and ### *P*<0.001

Serum levels of IL-4, IFN-γ, and IFN-γ/IL-4 ratio were studied in asthmatic rats after transplantation of c-kit^+^ and c-kit^-^ cells. ELISA assay showed a prominent difference in the levels of these cytokines in OVA-sensitized rats compared with the control rats. These data confirmed the pro-inflammatory status in asthmatic rats induced by OVA challenge. Compared with healthy control rats, we found significantly higher levels of IL-4 and reduction of INF-γ and IFN-γ/IL-4 ratio in asthmatic rats (*P*<0.001 to *P*<0.01; Figures 4a, b, and c). Interestingly, a significant decrease in IL-4 level coincided with increased INF-γ level, and the IFN-γ/IL-4 ratio was notified in asthmatic rats that received c-kit^+ ^cells compared with other sensitized rats (*P*<0.001 to *P*<0.01; Figures 4a, b, and c). No significant differences were found in the serum levels of IL-4, IFN-γ, and IFN-γ/IL-4 ratios in rats from A and A+ c-kit^- ^groups.

***Local administration of c-kit*** ^+^ ***cells*** ***returned the expression of miRNA-126 and miRNA-133 in asthmatic rats to the normal levels***

**Figure 5:**
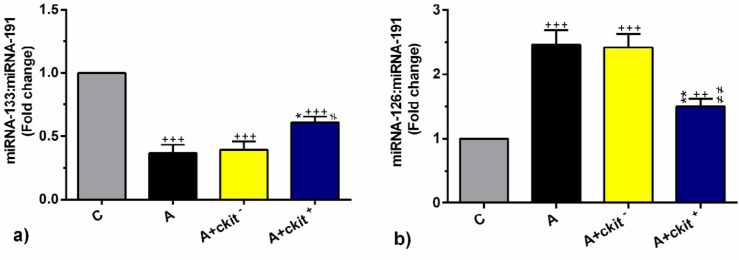
Real-time analysis of miRNA-126(a) and miRNA-133(b) expressions in the lungs of control group (C), sensitized group (A), sensitized animals received c-kit- cells (A + c-kit- group), sensitized animals received c-kit+ cells (A + c-kit+ group) (for each group, n=6). Bars represent the mean±SEM. Statistical differences between control and different groups: ++; *P*<0.01 and +++; *P*<0.001. Statistical differences between A+ c-kit+ and A+ c-kit- vs A group: *; *P*<0.05 and **; *P*<0. 01. Statistical differences between A+ c-kit+ and A+ c-kit- groups: #; *P*<0.05, ##; *P*<0. 01

Real-time PCR analysis showed that miRNA-133 was down-regulated significantly in all sensitized rats in comparison with the control group (*P*<0.001, Figure 5A). There was a significant increase in the expression of miRNA-133 in rats from the A+ c-kit^+^ group as compared with other sensitized rats (*P*<0.05, Figure 5a). However, non-significant differences were found in the values of miRNA-133 from A and A+ c-kit^-^ groups (Figure 5a). In contrast to miRNA-133 levels, the expression of miRNA-126 was significantly increased after asthmatic induction as compared with control rats (*P*<0.001 to *P*<0.01, Figure 5a). Transplantation of c-kit^+ ^cells was shown to prominently decrease the expression of miRNA-126 in A+ c-kit^+^ group in comparison with other sensitized rats (*P*<0.01, Figure 5b). No statistically significant differences were found in the expression of miRNA-126 between A and A+ c-kit^-^ groups (Figure 5b). These data demonstrated that the transplantation of c-kit^+^ cells, but not c-kit^- ^cells, has the potential to decrease asthmatic changes by modulation of miRNA-126 and miRNA-133.

***Transplanted c-Kit*** ^+^ ***cells alleviated the progression of asthmatic pathologies***

**Figure 6:**
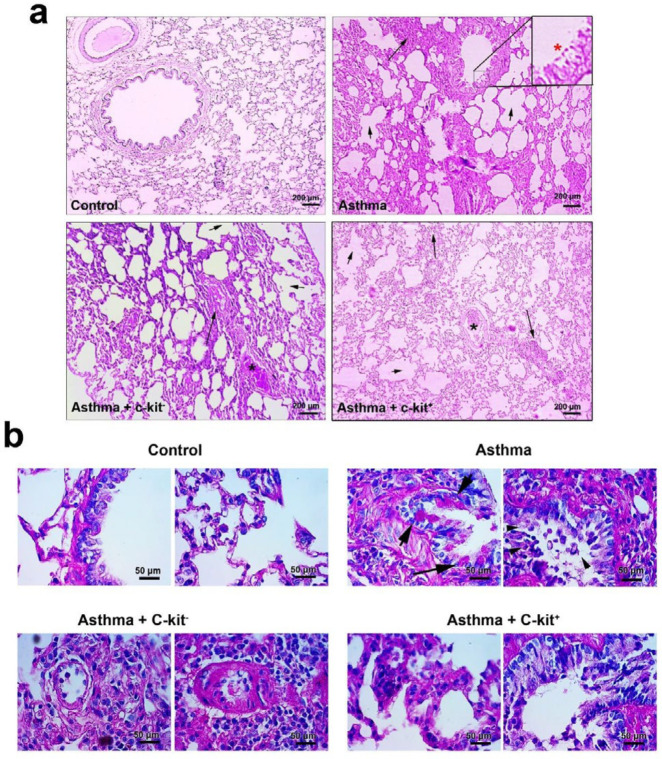
Bright-field images from pulmonary tissue sections stained with H&E (a). Normal lung tissue (group C). Hyperemia, immune cells infiltration, emphysema, and interstitial pneumonitis were seen in rats from groups A and A + c-kit-. In contrast, the transplantation of c-kit+ cells in asthmatic rats caused a slight decrease in immune cell infiltration and interstitial pneumonitis. The extent and intensity of other pathological features were also improved in sensitized rats receiving c-kit+ (A + c-kit+ group). Black Asterisks: Hyperemia; Long arrows: Interstitial pneumonitis; Short arrows: Emphysema; Red asterisks: Epithelial cells injury. PAS solution (b). PAS staining revealed the accumulation of glycosylated component in the asthmatic niche compared with the control group. In the A group, there are a lot of goblet cells in the bronchioles epithelial cell layer filled with the glycosylated proteins, showing increased mucus production in the A group (arrows). High magnification imaging revealed the detachment of the epithelial cell layer and infiltration of immune cells into pulmonary conduits (arrowhead). The transplantation of positive C-kit cells, but not negative C-kit cells, could decrease the accumulation of glycosylated components and immune cell recruitment to the asthmatic niche

The pattern of chronic pathological changes confirmed the efficiency of our protocol in the induction of asthma in the rat model (Figure 6, Table 2). Pathological injuries in the lung tissues of all sensitized groups were significantly higher than in the C group (*P*<0.001 to *P*<0.05). Transplantation of c-kit^+^ cells caused a decrease in all pathological indices compared with A and A + c-kit^- ^groups (*P*<0.001 to *P*<0.01). The score of pathological features in the A + c-kit^-^ group was similar to scores obtained from the A group (Figure 6a, Table 2). PAS staining revealed the existence of goblet cell proliferation, filled with the glycosylated protein inside the cytoplasm, coincided with the detachment of the bronchial epithelial cell layer. We also found the infiltration of inflammatory cells and isolated epithelial cells inside the bronchiolar conduits. Enhanced polysaccharide content was also detected in the context of pulmonary parenchyma which could be related to the existence of recruited immune cells as well as pneumonocyte proliferation. In the group receiving C-kit positive cells, but not C-kit negative cells, we found a prominent decrease in the PAS-stained intensity and decrease of goblet cells in the epithelial layer (Figure 6b).

## Discussion

Careful insight into patients that had undergone cell therapy and trials revealed the necessity for the discovery of reliable cell sources with regenerative potential that could circumvent the pitfalls and incomplete regeneration of injured tissues to inspire deep and secure sense in the target population (21). Although most experiments showed the therapeutic effects of stem cells, mechanisms beyond regeneration have as yet been tremendously neglected (9). To the best of our knowledge, the critical role of genetic players needs to be elucidated in relation to the paracrine activity of transplant cells particularly stem cells. Most notably, c-kit^+ ^cells belonging to stem cells have been shown to possess a unique regenerative capacity and immunomodulatory effects in animal cardiac tissues (22-25). However, experiments and therapies based on c-kit^+^ cells for airway inflammation are still in their infancy (26). The current study targets revealing the correlation between the anti-inflammatory effects of c-kit positive cells and distinct miRNAs contributing to the therapeutic paracrine outcome. The study and monitoring of the miRNA expression pattern is a sophisticated movement to dictate specific cell behavior or the ultimate regenerative potential in asthmatic lungs (16). Considering unique anatomical property and microstructural features of pulmonary tissue, target cells could be administered either via intratracheal or systemic routes (17). To ascertain an efficient cell homing to the pulmonary niche, we selected local intratracheal administration of bone marrow c-kit^+^ cells in the asthmatic rats (17). 

As expected, we successfully induced asthma in rats evident by pathological features and changes in the systemic levels of IL-4 and INF-γ (27). By transplanting c-kit^+^ cells, the intensity of pathological features and production of the above-mentioned cytokines were changed and reached near-to-control levels. Based on the previous data, the promotion of Th1/2 imbalance with enhanced Th2 activity exacerbated allergic pulmonary responses by releasing multiple cytokines IL-4, -5, and -13 (28). Therefore, regulation of Th2 activity could be touted as a strategic approach in the alleviation of asthmatic complications. Considering the close correlation of asthmatic changes with Th1/2 imbalance, it is logical to mention that the reduction of pathological changes in the c-kit^+^ cells group correlates with clonal expansion and activity of specific Th subpopulation. Consistent with our results, Spaziano and co-workers showed the intratracheal administration of 5 × 10^4 ^murine pulmonary c-kit cells had the potential to suppress the production of cytokines IL-4, -5, and -13 with polarization of macrophages to M2-like phenotype orchestrated possibly in paracrine and/or juxtacrine manner. Besides, the local content of IL-10 was also increased post-c-kit transplantation(26). The advantage of the current experiment is to investigate the potent anti-asthmatic activity of bone marrow c-kit cells (3 × 10^5^ cells) compared with local c-kit lineage and to highlight the underlying role of distinct mi-RNAs in pulmonary tissue. As the number of pulmonary-specific progenitor cells is trivial in lungs compared with bone marrow microenvironment, and there are serious ethical issues regarding isolation of these cells from pulmonary tissue, the use of bone marrow cells is more applicable in human medicine compared with c-kit from other sources (29). 

miRNAs are currently accepted as potent biomarkers in the diagnosis and treatment and follow-up of asthmatic subjects (16). As such, miRNA-126 and -133 play critical roles in the pathogenesis and dynamics of asthma (16). Based on our data, the injection of c-kit^+^ cells adjusted the transcription of these miRNAs to the normal levels. These changes were in accordance with the decrease of the Th2 subtype. The correlation of miRNA-126 and -133 with production of pro-inflammatory cytokines was previously determined in asthmatic subjects (30, 31). Previous data showed that the decrease of miRNA-126 *per se *diminished the recruitment of eosinophils to the asthmatic niche (30). The decrease of miRNA-133 stimulates bronchiolar smooth muscle cells relation via engaging the Rho signaling pathway (31). Changes in the clonal activity of distinct T helper type and regulation of miRNA-126 and -133 expressions in rats receiving c-kit cells showed the potency of c-kit marker harboring cells in the control of asthma pathogenesis via miRNAs element. It seems logical to mention that cells belonging to c-kit negative lineages are oriented to functional maturation with directed immune reaction activities. Therefore, these cells could possibly, but not completely, lose regenerative properties in response to different stimuli compared with the c-kit positive cells. The introduction of c-kit negative cells with limited regenerative capacity not only alleviated the asthmatic changes but also could exacerbate immunological responses in the inflamed tissues in response to multiple arrays of cytokines. There are some limitations related to this study. We suggest that monitoring the activity of miRNA-126 and -133 target genes could give us helpful information about mechanisms beyond c-kit application in the asthmatic rats.

**Table 1:** Primer set list used for miRNAs

**Gene name**	**Gene bank accession no.**	**Target sequence** ^a^
miR-126	MIMAT0002957	UCGUACCGUGAGUAAUAAUGC
miR-133	MIMAT0017124	AGCUGGUAAAAUGGAACCAAAU
miR-191	MIMAT0000866	CAACGGAAUCCCAAAAGCAGCUG

^a^Sequences were derived from miRBase (http://www.mirbase.org)

MACS: magnetic activated Cell sorting

**Table 2:** Pathological scores in the lungs of control group (C), sensitized group (A), sensitized animals receiving c-kit- cells (A + c-kit- group), sensitized animals receiving c-kit+ cells (A + c-kit+ group) (for each group, n=6)

Pathological findings	Scores in groups (for each group, n = 6)			
	(Minimum-Maximum)			
	C	A	A + c-kit^-^	A + c-kit^+ ^
Hyperemia	(1-0)	(1-3) +++	(2-3) +++	(1-2) + * #
Interstitial pneumonitis	(0-0)	(2-3) +++	(2-3) +++	(1-2) + ** ##
Emphysema	(0-0)	(2-3) +++	(1-3) +++	(1-2) ++ ** #
Epithelial cells injury	(0-0)	(2-3) +++	(1-3) ++	(0-2) + **

Statistical differences between control and different groups: ++; *P*<0.01 and +++; *P*<0.001. Statistical differences between A+ c-kit+ and A+ c-kit- vs A group: *; *P*<0.05 and **; *P*<0.01. Statistical differences between A+ c-kit+ and A+ c-kit- groups: #; *P*<0.05, ##; *P*<0. 01. The lowest–highest pathological values in each group were shown between the parentheses

## Conclusion

Overall, the local administration of c-kit^+^ cells could alleviate the asthmatic pathology possibly by the control of miRNA-126 and -133. No obvious immunomodulatory effects were found in rats receiving c-kit^-^ cells.
